# Combinatorial Computational Approaches to Identify Tetracycline Derivatives as Flavivirus Inhibitors

**DOI:** 10.1371/journal.pone.0000428

**Published:** 2007-05-09

**Authors:** Jinn-Moon Yang, Yan-Fu Chen, Yu-Yin Tu, Kuei-Rong Yen, Yun-Liang Yang

**Affiliations:** 1 Department of Biological Science and Technology, National Chiao Tung University, Hsinchu, Taiwan; 2 Institute of Bioinformatics, National Chiao Tung University, Hsinchu, Taiwan; Vanderbilt University, United States of America

## Abstract

Limited structural information of drug targets, cellular toxicity possessed by lead compounds, and large amounts of potential leads are the major issues facing the design-oriented approach of discovering new leads. In an attempt to tackle these issues, we have developed a process of virtual screening based on the observation that conformational rearrangements of the dengue virus envelope protein are essential for the mediation of viral entry into host cells via membrane fusion. Screening was based solely on the structural information of the Dengue virus envelope protein and was focused on a target site that is presumably important for the conformational rearrangements necessary for viral entry. To circumvent the issue of lead compound toxicity, we performed screening based on molecular docking using structural databases of medical compounds. To enhance the identification of hits, we further categorized and selected candidates according to their novel structural characteristics. Finally, the selected candidates were subjected to a biological validation assay to assess inhibition of Dengue virus propagation in mammalian host cells using a plaque formation assay. Among the 10 compounds examined, rolitetracycline and doxycycline significantly inhibited plaque formation, demonstrating their inhibitory effect on dengue virus propagation. Both compounds were tetracycline derivatives with IC_50_s estimated to be 67.1 µM and 55.6 µM, respectively. Their docked conformations displayed common hydrophobic interactions with critical residues that affected membrane fusion during viral entry. These interactions will therefore position the tetracyclic ring moieties of both inhibitors to bind firmly to the target and, subsequently, disrupt conformational rearrangement and block viral entry. This process can be applied to other drug targets in which conformational rearrangement is critical to function.

## Introduction

The Dengue virus (DV) belongs to the Flavivirus family and has become a major threat to public health globally, especially in tropical and subtropical areas, due to the increases in population density and environmental changes. There are approximately 2.5 billion people who live under the shadow of DV infection. Other well-known Flaviviruses include yellow fever virus, Japanese encephalitis virus, West Nile virus [1], [2], and Murray Valley encephalitis virus [3]. The Dengue virus has four serotypes and is transmitted by *Aedes* mosquitoes. Patients with DV infection show various clinical symptoms that range from no significant illness or mild fever to life-threatening Dengue hemorrhagic fever (DHF) and Dengue shock syndrome (DSS) [4]. Currently, only supportive treatments are available. Although considerable research has been directed towards the development of a safe and effective DV vaccine since the mid-20th century, there are no approved commercial products available [5]. Therefore, to combat DV and other related viral diseases, it is advisable to develop novel strategies for discovering new antiviral agents. Recent progress in the biology has brought with it many protein structures for virtual screening (VS) as drug targets [6]–[9]. However, without a previously validated target site on the targeted protein as a reference point, the number of lead candidates obtained from this type of screening is very large. Cellular toxicity further complicates biological activity assays as well. Therefore, the utilization of VS is somewhat hindered by the processes that follow, namely, the labor-intense, time-consuming verification process and the toxicity assays required for processing large amounts of lead candidates. Here, in an attempt to devise a less resource-demanding screening process, we have focused on computational approaches that are solely based on the structures of a designated region of the target protein. Then, we performed VS on a set of medical compounds because we recognized that using medical compounds could potentially minimize cellular toxicity. To reduce the number of lead candidates, we further refined the VS output by structural clustering for the identification of novel structural characteristics. Compounds with novel structures were then subjected to a biological assay to validate their activities. In summary, we sacrificed the diversity of leads in exchange for the efficiency of screening.

The DV envelope (E) protein is 495 amino acids in length, forms oligomers, and, along with the M protein, constitutes most of the accessible virion surface that is covered by the envelope membrane. The E protein is responsible for activating “membrane fusion”, the central molecular event during the entry of enveloped RNA viruses into host cells. The Dengue virus enters a host cell when the E protein binds to the virus receptor [10] on the host cell surface and activates its conformational rearrangement, causing the E protein in its dimeric pre-fusion form to transform into a trimeric post-fusion structure. This essentially irreversible conformational change induces the fusion between the viral envelope membrane and the host cell membrane [11], allowing entry to be completed. In short, the DV E protein mediates host cell binding and is essential for infection via a conformation-induced membrane fusion event between the host cell and the virion. In addition, it is also the primary antigen that induces protective immunity and the major antigen for virus neutralization [10].

**Figure 1:**
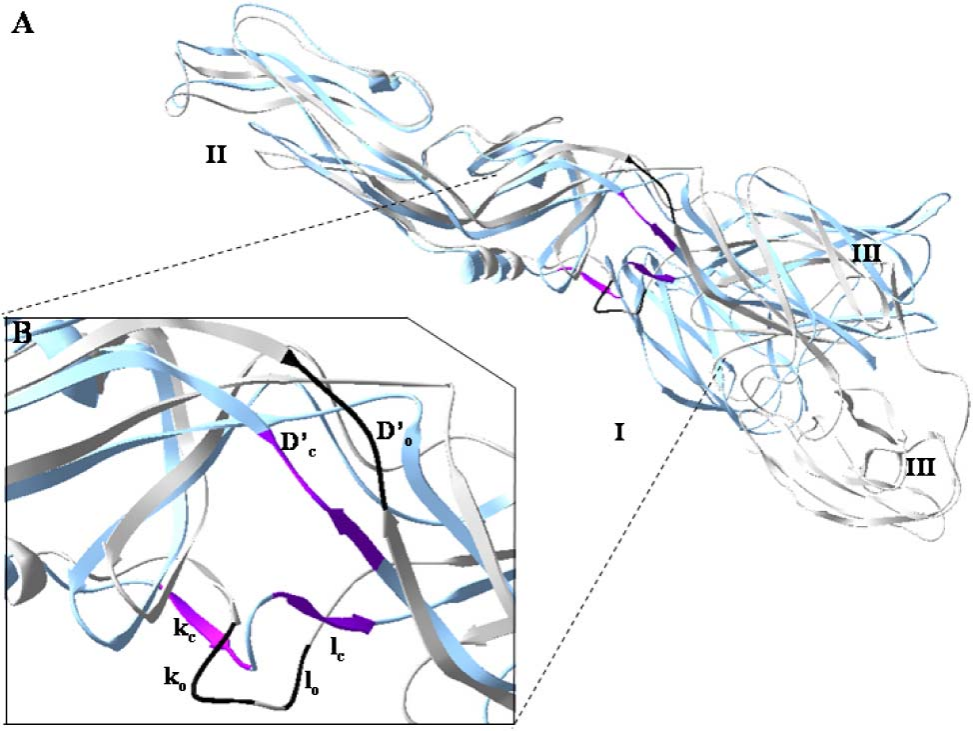
Pre-fusion (PDB code 1oke) and post-fusion (PDB code 1ok8) conformations of Dengue virus E protein and the ligand-binding pocket for virtual screening. (A) Dengue virus E protein structures in pre-fusion (gray) and post-fusion (blue) states and the position of the binding regions (black strand representing *D′*, *k*, *l* in pre-fusion state, colored strand representing the post-fusion state). (B) The conformation rearrangement of the binding areas. Higher-order structures and domains I, II, and III are defined according to Modis et al. [11].

The crystal structures of the E protein of DV type 2 in both the presence (pre-fusion) and absence (post-fusion) of a bound ligand were deposited in the Protein Data Bank {PDB codes 1oke [5] and 1ok8 [11], respectively; Figure 1). The key difference between these two structures is a local rearrangement of the “*kl*” β-hairpin (residues 268–280) and the concomitant opening up of a hydrophobic pocket for ligand binding. For example, the detergent *n*-octyl-β-D-glucoside (BOG) can occupy this pocket [11]. Mutations that affect the pH threshold for membrane fusion have also been mapped to this hydrophobic pocket [12], [13]. Therefore, Modis et al. proposed that this pocket was a hinge point in the fusion-activating conformational change and suggested that it could be a target site for the development of fusion inhibitors [5], [11] that could disrupt or even block the correct conformational changes necessary for DV entry. This concept made the utilization of structure-based VS to identify inhibitors of DV infection plausible.

Therefore, in this study, a well-developed docking tool, GEMDOCK [14]–[17], was utilized to perform VS on the Comprehensive Medicinal Chemistry (CMC) database for substances that could dock in this hydrophobic pocket of E proteins [5]. These compounds were then selectively tested, based on distinct structural characteristics, for the inhibition of DV propagation. We have now successfully identified two tetracycline derivatives [18], [19] that displayed significant inhibitory effects on the propagation of the DV type 2 PL046 strain in cell cultures. According to the docked conformations of these two active, and of two inactive tetracycline-derived compounds, we have proposed a model for the inhibition of DV E protein conformational change, which may provide a future direction for lead compound optimization.

## Results

### Virtual screening for inhibitors of the E protein

**Figure 2:**
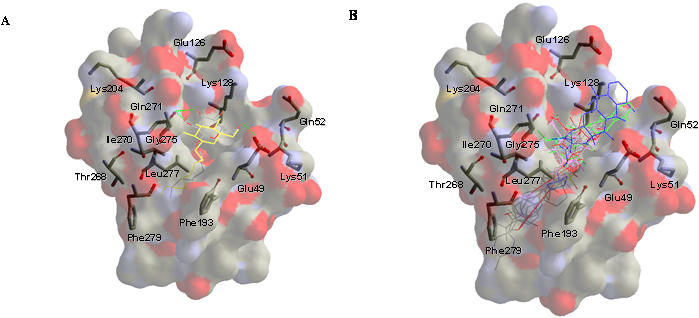
Docked conformations of the candidate compounds in the BOG binding site according to GEMDOCK. The residues affecting the pH threshold of fusion are indicated. (A) The crystal conformation is shown in the CPK model (i.e., oxygen in red, nitrogen in blue, and carbon in gray), and the docked conformation of the BOG is shown in yellow. The formation of the hydrogen bonds is shown by a green dashed line. The RMSD of the conformations is 1.20 Å, and both pre-fusion and post-fusion conformations form hydrogen bonds with Glu49 and Gln271. (B) The docked conformations of the 10 selected compounds are shown. The four tetracycline derivatives are colored (doxycycline in green, rolitetracycline in blue, tetracycline in orange, and oxytetracycline in red). The inhibitory compounds (doxycycline and rolitetracycline) are docked in the vicinity of residues Thr48, Glu49, Ala50, Lys51, and Gln52.

To assess the VS program, we first evaluated the docking accuracy of GEMDOCK for the DV E protein by docking the detergent ligand (BOG) into the binding site. The docked conformation of BOG (Figure 2A) with the lowest scoring value was compared with the crystal structure of BOG based on the root mean square deviation (RMSD) of heavy atoms. The average RMSD of 10 independent runs was less than 1.20 Å. Molecular recognition of the E protein was also investigated to determine the constraints of the ligand and pharmacophore preferences during the VS. This detergent-binding pocket, located at the juxtaposition of domains I and II of the E protein, is hydrophobic in the pocket [5], [11] and hydrophilic on both sides of the protein surface.

GEMDOCK was then used to perform VS on the DV E protein using a screening set from the CMC database that contained 5,331 molecules between 200 and 800 Daltons. Since the binding site of the DV E protein is hydrophobic, we set the electrostatic constraint, based on the upper bound number of charged atoms, to 0 and the hydrophilic constraint, based on the upper bound fraction of polar atoms, to 0.3 (equation 4 in Materials and Methods) to reduce the effects of GEMDOCK bias toward charged polar compounds. The ligand preference served as a hydrophilic filter and penalized compounds that had high hydrophilicity. Since our previous studies indicated that the ligand and pharmacophore preferences contributed to improvements in the enrichment of VS [14], [16], we used the scoring values of both the empirical scoring function and pharmacophore-based scoring function as ranking conditions to identify inhibitor candidates of the DV E protein.

**Figure 3:**
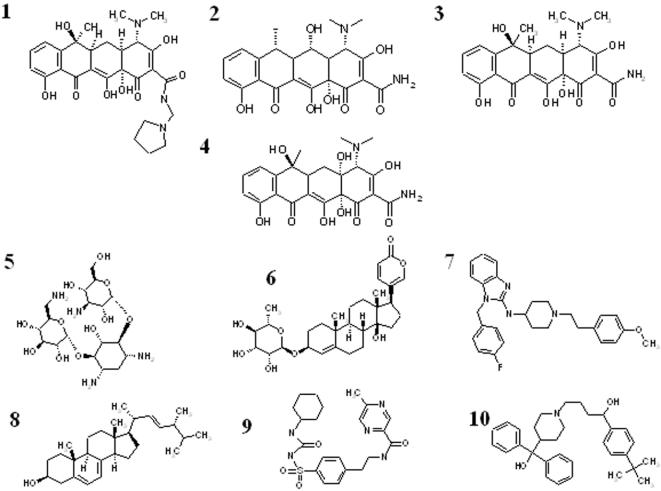
The ten compounds selected for competitive blocking assay of DV propagation. 1, rolitetracycline; 2, doxycycline; 3, tetracycline, 4, oxytetracycline; 5, kanamycin; 6, proscillaridin; 7, astemizole; 8, ergosterol; 9, glipizide; 10, terfenadine.

We selected the top-ranking 3% of compounds (∼173 compounds, see Appendix S1 in supporting material) for further analyses to enrich the hit rate after GEMDOCK screening. These candidate compounds were then clustered using a hierarchical cluster method based on both their two-dimensional compound structures and protein-ligand interactions [20], [21], similar to Jain's work [22]. Here, atomic environments [20], [21] were used to represent the two-dimensional compound structure for measurements of compound similarities and the protein-ligand interactions were used for the identification of docked positions and hot spots. Based on structural similarities, docked positions, protein-ligand interactions, and the limitations of commercial availability, two groups of structures (Figure 3) distinguished themselves for use in the *in vivo* plaque formation assay for their potential inhibitory effects on DV propagation in cultured cells. One group consisted of two tetracycline derivatives (tetracycline and rolitetracycline) and the other group consisted of connected ring structures with additional flexibility. To enrich possible hits, two more tetracycline derivatives (doxycycline and oxytetracycline) under similar atomic conditions were also included for the biological activity assay. Docked conformations of these selected compounds are shown in Figure 2B and the four tetracycline derivatives are indicated as blue (rolitetracycline), green (doxycycline), orange (tetracycline), and red (oxytetracycline). As shown in Figure 2A, BOG is docked in the pocket and is situated centrally among Gly275, Lys128, Leu277, and Gln52. All ten selected candidate compounds were able to dock in the pocket at various locations (Figure 2B).

### *In vivo* plaque formation assay

**Figure 4:**
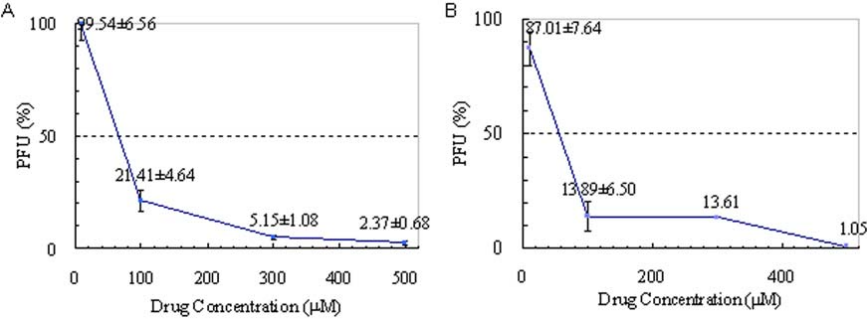
Effects of (A) doxycycline and (B) rolitetracycline on Dengue virus type 2 plaque formation using BHK-21 mammalian cells. The IC_50_ values of rolitetracycline and doxytetracycline are 67.1 µM and 55.6 µM, respectively. The *x* axis shows the percentage of the amount of plaque formation compared with control. The *y* axis denotes the drug concentration.

**Figure 5:**
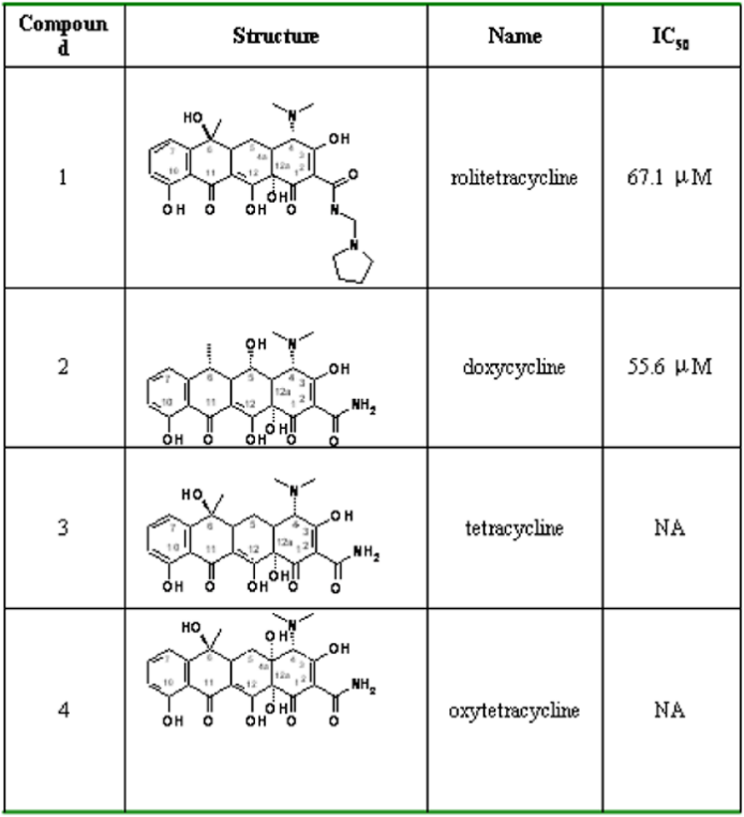
Chemical Structures and IC_50_s for the tetracycline derivatives. Name, chemical name; IC_50, _the half maximal inhibitory concentration; NA, not applicable.

To assess whether those individual compounds obtained by screening could indeed affect the propagation of Dengue virus replication as predicted, different concentrations of the compounds were added separately to cultures of BHK-21 cells, followed immediately by the addition of the DV type 2 PL046 strain at a fixed number of plaque forming units (PFUs). If the compounds bind to the E proteins as predicted, they may interfere with the interactions between the E protein and the host surface receptor, particularly with the E protein conformational change that is necessary to activate viral entry. This inhibition would reduce the frequency of DV infection in BHK-21 cells. Since every successful infection leads to the formation of a plaque, the number of plaques on the assay plate indicates the number of infection events. As a fixed number of PFUs was originally added to the culture, the reduction in the number of plaques reflected the portion of the virion infection that was inhibited by the presence of the particular compound. Therefore, using the number of PFUs from the culture plates added only media (no compounds) as 100%, the relative percentage of the PFUs from the culture plates with compounds was calculated. Of the 10 compounds, rolitetracycline and doxytetracycline (Figure 4) showed dramatic inhibitory effects on DV propagation. In addition, another compound, oxethazaine, also showed mild inhibition. There was a 12% reduction (down to 88% from that of control) in the PFUs when the concentration of oxethazaine in the culture was increased from 200 µM to 500 µM. For 10 µM rolitetracycline, there was almost no effect on DV plaque formation. But as the concentration of rolitetracycline was increased, there were significant inhibitory effects on DV propagation. Compared with controls, there were only 20% of the PFUs remaining at 100 µM and approximately 5% at 300 µM, yielding an estimated IC_50_ value of 67.1 µM (Figure 4). At 500 µM, there were less than 3% PFUs remaining. As for doxycycline, 87% of the PFUs were retained at 10 µM and 14% were retained at 100 µM. When the concentration of doxycycline reached 500 µM, there was only 1% of the PFUs remaining, yielding an IC_50_ value of 55.6 µM (Figure 4). Interestingly, neither tetracycline nor oxytetracycline showed an effect on DV propagation at concentrations ranging from 10 µM to 10 mM (data not shown), even though they share the tetracyclic ring structure with both rolitetracycline and doxycycline. Figure 5 shows the molecular structures and IC_50_ values of the tetracycline derivatives. These compounds had no cellular toxicity effects within the range of concentrations tested as judged from both cellular morphology and growth with one possible exception. When the culture contained doxycycline at a concentration of 500 µm or greater, the cell density appeared to be reduced. We further tested the effects of those compounds by the addition of 500 µm of individual compounds together with or at intervals after the addition of DV to the cultured cells. The results revealed that when 500 µM of either one of the active compounds were added to the cell cultures together with a fixed number of PFUs, the number of plaques formed was approximately 3% or less compared with controls, whereas approximately 75% of the PFUs remained when the compound was added 2 hours after the presence of viruses in the cell culture. Therefore, the inhibitory effect is time-dependent. That is, if the compounds are added after sufficient time was allowed for the infection to proceed, the compounds lost potency.

### Computational analysis of inhibitor-E protein interactions

**Figure 6:**
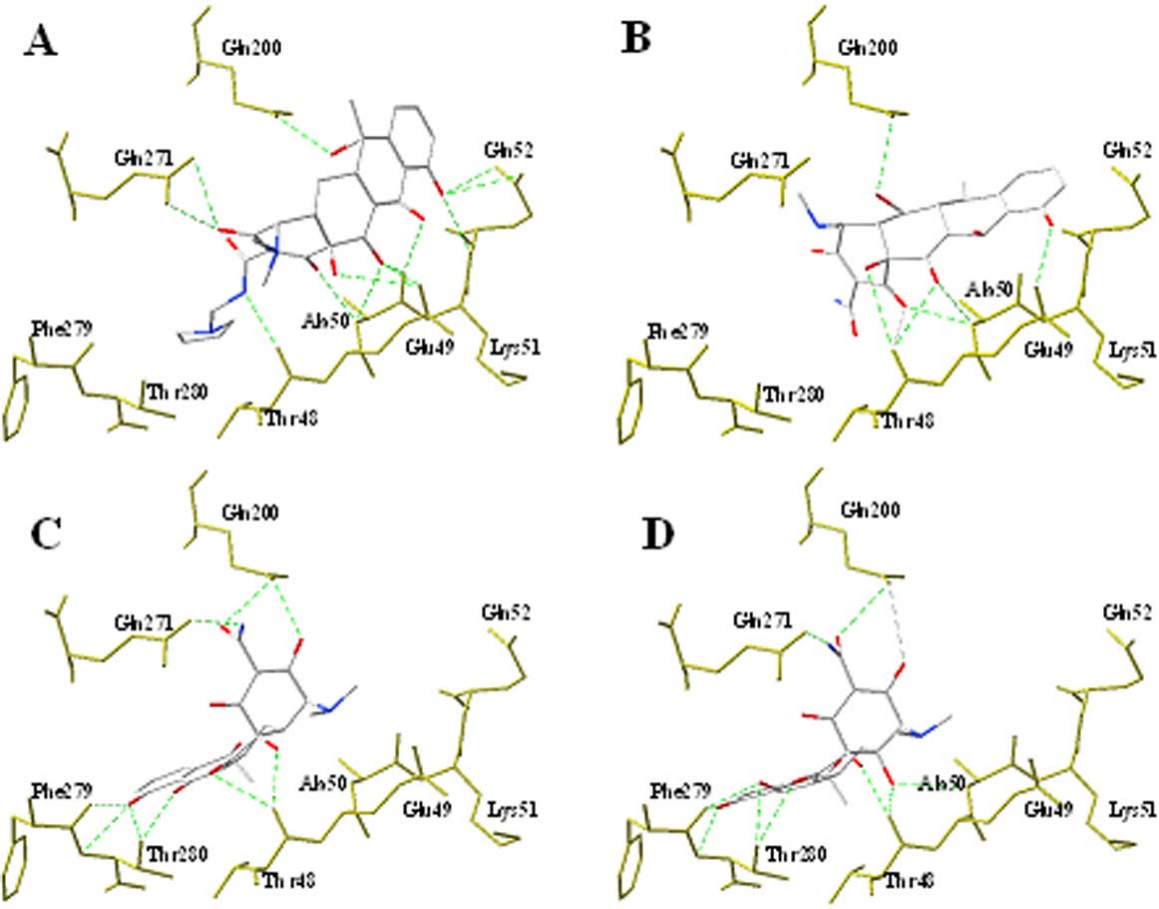
Docked conformations and hydrogen bonding of rolitetracycline (A), doxycycline (B), tetracycline (C), and oxytetracycline (D) to the BOG binding site of the DV E protein. Atoms of the E protein are shown in yellow and compound ligands are shown in CPK model. The hydrogen bonds are represented as green dashed lines. Not all residues are displayed for the sake of clarity. Thr48, Glu49, Ala50, Lys51, and Gln52 are in the *D′_0_* segment (Figure 1B), while Gln271 and Phe279 are in the *k_o_* and *l_o_* segments, respectively.

**Figure 7:**
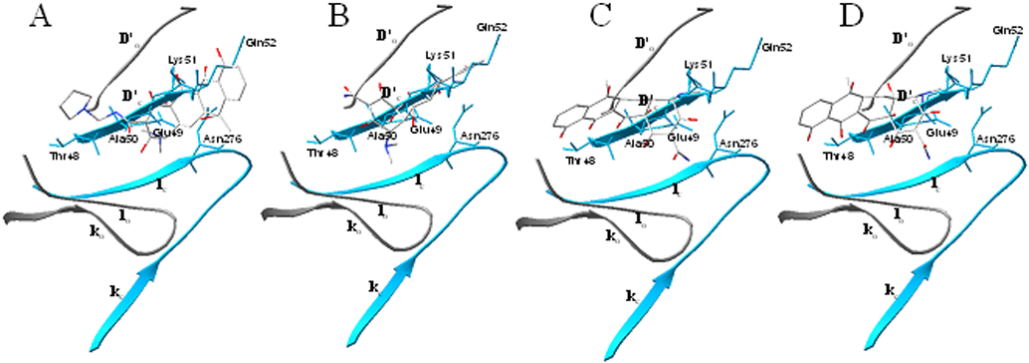
Docked conformations of the four tetracycline derivatives related to residues 48–52 in the BOG binding site in the pre-fusion (gray) and post-fusion (blue) states. (A) rolitetracycline, (B) doxycycline, (C) tetracycline, and (D) oxytetracycline. Atoms within the compounds are displayed using the CPK model (oxygen in red, nitrogen in blue, and carbon in gray). The side chains of certain residues that overlap with the compounds are displayed. The segments *D*′*_o_*, *k_o_*, and *l_o_* are present in the pre-fusion conformation, while *D*′*_c_*, *k_c_*, and *l_c_* are present in the post-fusion conformation.

The docked conformations of the two tetracycline-derived inhibitors were consistently different from those of the eight non-inhibitory compounds according to the computation program used (Figures 2B, 6, and 7). The inhibitors, doxytetracycline (green) and rolitetracycline (blue) were docked on the outside of the binding pocket and extended into the pocket while the non-inhibitory compounds (CPK model) were docked inside the pocket (Figure 2B). The inhibitors were docked between the D and I segments of which the conformations significantly differed between the pre-fusion and post-fusion forms (Figures 1B and 7). In fact, the inhibitors were docked very close to or at the *D′c* space and their tetracyclic structure was beneath *D′c* between *D′c* and *Ic* (Figures 1B and 7). Those compounds could not only cause steric hindrance by their structures *per se* but could also interact with the polypeptide stretch of residues 48 to 52 via their functional groups (Figure 6 A–B and 7 A–B). The residues in the stretch, formed by Thr48, Glu49, Ala50, Lys51, and Gln52 and several others in the vicinity, were shown to affect the pH-dependent membrane fusion process [5], [12], [22]–[26]. The locations of these residues in the crystal model are indicated in Figure 2. On the other hand, the two inactive tetracycline compounds were docked further away from the *D′c* space and their tetracyclic structures were localized above *D′o* and *D′c*. Hence, they would not create steric hindrance during the switch from *D′o* to *D′c* (Figure 7, C–D). Figure 6 shows the hydrogen-bonding networks and orientations of the four tetracycline derivatives with regard to the E protein in the pre-fusion form. We also observed that the derivatives could be divided into two groups by their docked locations. Those with inhibitory effects, rolitetracycline (Figure 6A) and doxycycline (Figure 6B), were docked in positions near residues 48–52 and formed hydrogen-bonding networks with residues Thr48, Glu49, Ala50, Lys51, and Gln52, as well as Gln271 and Gln200. Conversely, the other two compounds, tetracycline (Figure 6C) and oxytetracycline (Figure 6D), formed hydrogen bonds primarily with residues Thr280, Phe279, Gln271, and Gln200. Tetracycline interacted with the 48–52 stretch only at Thr48 while oxytetracycline interacted with residues Thr48 and Ala50 and both appeared to prefer Phe279 and Thr280. In addition, the inhibitors bound to opposite sides of the surrounding wall (residues 48–52 vs. residues 200 and 271) of the binding pocket and extended their structures centrally into the pocket, while the non-inhibitors bound entirely to one side of the pocket (residues 200, 271, 279, 280, and 48) (Figures 2B and 6). Furthermore, GEMDOCK yielded lower binding energies for the two inhibitors than for the inactive compounds. The energy minimization process performed by SYBYL 6.9 also indicated that the predicted inhibitor complexes had lower energies than the non-inhibitors. The energies of rolitetracycline, doxytetracycline, tetracycline, and oxytetracycline were −395.2, −398.7, −356.8, and −371.8 kcal/mol according to SYBYL 6.9.

## Discussion

For the eradication of infections caused by enveloped viruses, the identification of compounds that can block the function of viral envelope proteins to prevent viral entry has been a long-standing idea in the field. However, mass screening is usually considered too costly and, for the more design-oriented approaches, VS with limited information tends to yield too many candidates for biological activity assays and is usually further complicated by the cellular toxicity possessed by many of the candidates. Here, we have devised a scheme in which VS focused on both the steric hindrance and atomic environment between the compounds and the targeted E protein to minimize the number of candidates. And, to further reduce the number of candidates, instead of using the whole E protein structure as the target for VS, we isolated the small region around a chosen target site to serve as the target. Although this approach may limit the diversity of the potential leads due to the diminished choices of possible target sites for VS, we believe that this methodology will, in fact, help to enhance the chance of a successful hit because the program can screen many more compounds with more thoroughness within the same time frame. In this study, we chose the hydrophobic detergent-binding pocket reported by Modis et al. as the target [5], [11]. This putative detergent-binding site is located in the E protein between domains I and II, which are the key structural elements involved in the pH-induced conformational rearrangement that is essential for DV entry. Therefore, a suitable target for small-molecule inhibitors would be the blockade of the conformational change of the E protein and, subsequently, the inhibition of viral-host membrane fusion, which would interrupt viral entry and block infection [5], [11]. Additionally, mutations in the DV E protein mapped to this pocket indeed affect the pH threshold of fusion [5], [12], [22]–[26]. In short, based on the structural study of Modis et al. [5], [11], we developed a VS process and was successful in applying it to the identification of lead compounds that inhibit DV propagation. After computation, there were only ten non-toxic candidate compounds that required validation by biological activity assays.

It is very interesting that in this study, even though tetracycline and oxytetracycline share similar tetracyclic ring structures with both rolitetracycline and doxycycline, they are not inhibitory (Figure 5). Tetracycline derivatives are a group of broad-spectrum antibiotics and were first discovered in the 1940s [19]. The mechanism of action of tetracycline and its derivatives on bacteria is via the inhibition of cellular protein synthesis by preventing the attachment of aminoacyl tRNAs to the ribosomal acceptor (A) site [18], [19], [27]. Those antibiotics consist of a linear, fused tetracyclic core to which a variety of functional groups are attached [19]. Tetracycline, in fact, contains the minimal common structure of the tetracycline-related molecules in this study. Therefore, this common structure *per se* does not possess the inhibitory effect on DV propagation. Instead, the substituted functional groups appear to confer anti-Dengue virus activity.

On the other hand, it is also possible that the active compounds affect the host cells instead of the virions. If this is the case, the compounds might inactivate a host cellular component that is essential for viral propagation. We believe that this scenario is unlikely since there are no significant differences in cellular morphology and growth, unless the function of such a cellular component, when compromised, affects only the viruses. Nonetheless, to test this hypothesis, we performed an assay in which the compounds were added to the cultures either together or 2 hours after the presence of viruses in the cell cultures. If the compounds were active against the viruses instead of the hosts, then adding them together to the culture should effectively block viral infection whereas the addition of the compounds 2 hours after the presence of viruses would not have the same effect since the viruses would have already proceeded through the entry event and infected the host cells. As expected, when the compounds were added together with the viruses, the PFUs were approximately 3% or less than controls, whereas those added two hours later were approximately 75% of control levels. Therefore, the compounds were less potent after the viruses had entered the host cells. Hence, these compounds most likely act upon a virus target site and only affect an event that occurs prior to the completion of viral entry.

Another possibility is that the compounds act on viral RNA at locations where the RNA structures are similar to the tetracycline-binding sites on the ribosomal RNAs or tRNAs of the bacteria. This possibility requires that these compounds can penetrate the virion structure to interact with the viral RNA to prevent viral entry while, simultaneously, they do not affect the viral RNAs inside the host cells when these same compounds are added two hours later. We believe this scenario is also highly unlikely. First, for the compounds to reach the viral RNAs in the virions, they would have to overcome the physical obstacle consisting of viral structural proteins. Second, if the viral RNAs are indeed the targets, the compounds should be effective regardless of the time of compound addition because they could still enter the host cells and bind the viral RNAs to disrupt replication.

As for the possibility that the compounds affect viral proteins other than the E protein, we believe that this scenario is also unlikely since the E protein is the only protein required for viral entry. However, we cannot rule out that the compounds may bind at sites other than our predicted locations on the E protein. To reveal the exact location of the compound-E protein interaction, it may be necessary to devise an experiment, such as co-crystallization of the protein and those compounds, in which the compounds can be labeled and traced at an atomic level so their exact docking locations can be identified relative to the binding pocket.

Nonetheless, we have conducted computational modeling in an attempt to provide a direction for future investigation. First, to assess the results of the binding of tetracycline derivates to the DV E protein, we compared the BOG binding sites of the DV E protein to the tetracycline-binding site on the tetracycline repressor, TetR. TetR regulates resistance to tetracycline in gram-negative bacteria. The tetracycline-binding site of the TetR protein has been defined and the structure determined by crystallography [28]. We found that the TetR protein shares similar characteristics with the E protein in the binding sites for the tetracycline derivatives. First, there is an appropriate volume in the binding sites. The volumes of the binding sites of various TetR crystals range from 359 Å^3^ to 495 Å^3^ whereas the BOG binding site on the E protein is 481 Å^3^, according to the tool program, Q-SiteFinder [29] (the first column of Table S1). Therefore, there is proper space for the tetracycline derivatives to fit into the BOG binding site. Second, there are hydrophobic surfaces in the pockets of both binding sites (Figure S1). Third, according to the results of a cross-docking test performed for TetR and the tetracycline derivatives (Table S1), the binding sites of the DV E protein and TetR permit the binding of the tetracycline derivatives. In addition, the hydrogen bonds formed between the tetracycline derivatives and the DV E protein are similar to those between TetR and the tetracycline-derived ligands (Table S2). Therefore, tetracycline derivatives should reasonably bind the BOG pocket of the DV E protein.

On the other hand, only two of the derivatives are inhibitory; therefore, the atomic details of the functional groups and the tetracyclic core must confer the inhibitory activity. Hence, we have analyzed the docked conformations and hydrogen bonding of the derivatives to assess the interaction between those compounds and the E protein. There are distinct differences between the effective and ineffective compounds (Figures 6, 7, and S2); the effective compounds have their tetracyclic cores positioned inside the pocket while their side chains form hydrogen bonds with the residues located on the opposite sides of the wall around the pocket and are capable of creating steric hindrance to the conformational alteration of the E protein. In contrast, the ineffective compounds form hydrogen bonds only with one side of the wall and their cores lean away from the pocket.

Next, on an atomic level, the predicted positions of the tetracycline derivatives with the E protein are shown in Figures 6 and 7. The fused tetracyclic rings of rolitetracycline and doxytetracycline bind along the *D′o* strand and occupy the *D′c* space of the E protein. The residues 48–52 are in the D segments. These compounds both interact mainly with Thr48, Glu49, Ala50, Gln200, and Gln271 through hydrogen bonds. Such a hydrogen-bonding network provides strong attraction forces to stabilize the binding of rolitetracycline and doxytetracycline to the *D′o* strand and the *kl* β-hairpin. In contrast, although these compounds have the same tetracyclic core structures, neither tetracycline nor oxytetracycline is inhibitory. Both compounds form hydrogen-bonding networks with Thr48, Gln200, Gln271, Phe279, and Thr280 (Figure 6); therefore, their tetracyclic rings are docked toward one side of the binding site and contact the surrounding hydrophobic residues via van der Waals interactions, which are very different from those of rolitetracycline and doxytetracycline.

During the process of E protein-host membrane fusion, the E protein structure is dramatically re-configured to allow the fusion peptide to properly interact with the host membrane. This event is marked by the rearrangement of the *kl* β-hairpin and the *D′o* segment (Thr48, Glu49, Ala50, Lys51, and Gln52) in the BOG binding site (Figures 1A and 6). The docked positions of the inhibitors suggest that they occupy the *D′c* and *kl* β-hairpin spaces in the post-fusion state and form a stable hydrogen-bonding network (Figures 6A, 6B, 7A and 7B). Therefore, these compounds block the rearrangement of the β-hairpin and *D′o* strand, and thereby block the rearrangement of domains II and I of the E protein during membrane fusion. Residues 48-52 are not only important to inhibitor binding but may also directly affect flavivirus membrane fusion. This hypothesis is consistent with previous reports that Gln52 may affect the pH threshold of fusion in flaviviruses [5].

Our study has presented a cost-effective and time-saving screening process that is based on limited structural information. We have successfully identified two novel tetracycline-derived inhibitors of the propagation of flavivirus DV type 2 PL046, by the computer-aided screening of the E protein structure followed by the biological assay validation of the candidate compounds in a cell culture system. These compounds may serve as the basis for the development of new treatments against Dengue virus infection. This procedure may be applied to other viral pathogens or for any other mechanism that involves specific conformational alterations for biological function. Our study also highlights the additional characteristics of certain tetracycline derivatives as effective inhibitors of DV propagation, which will allow further refinement of our screening program and potential medical application.

## Materials and Methods

The VS method generally encompasses four phases that are based on high-throughput molecular docking methods and the crystal structures of the target proteins. These phases include target protein preparation, compound database preparation, molecular docking, and post-docking analysis [6]. The preparation phases involve formatting the structural data from the target protein and compounds into acceptable forms for the docking program. Then, the method of molecular docking is employed to screen the compound library for potential leads that can dock onto the target protein, whereas post-docking analysis serves to enrich the hit rate.

### Preparations of the target protein and screening set

We prepared the compound set from the CMC database in May 2004 based on two criteria: molecular weights ranging between 200 and 800 Daltons and excluding compounds with multiple components. We eventually obtained a set of structures that consisted of 5,331 compounds. To reduce the complexity and running time of the computational program, we isolated the structure of the BOG binding pocket of the DV E protein (Figure 1B) in the BOG-bound conformation {PDB code 1oke [5]} and prepared it for the docking tools. The isolated area included amino acids enclosed within a 10-Å radius that centered on the bound ligand. The coordinates of the protein atoms were taken from the PDB for the screening process. GEMDOCK docked each compound in the screening set against this binding cavity and ranked each compound by the docked energy of the docked conformation. Then, those candidates were subjected to structural clustering [21]. According to the ranking, compound structures and the interactions between compounds and residues in the binding site were further selected for *in vivo* biological activity assays to assess their inhibitory effect on Dengue virus propagation in cell culture.

### Docking method and scoring function

Our previous work [14]–[17] showed that the docking accuracy of GEMDOCK was better than some well-known docking tools, such as GOLD [30] and FlexX [31], on a diverse data set of 100 protein-ligand complexes suggested by Jones et al. [30]. The accuracy of GEMDOCK was also better than GOLD, FlexX, and DOCK on screening the ligand database from Bissantz et al. [32] for TK [33] and the ER-antagonist receptor [16]. In this study, GEMDOCK parameters for flexible docking included the initial step sizes (σ = 0.8 and Ψ = 0.2), family competition length (*L* = 2), population size (*N* = 300), and recombination probability (*p_c_* = 0.3). For each ligand screened, GEMDOCK optimization was terminated either when the convergence was below a certain threshold value or when the iterations exceeded a maximal preset value of 60. For the latter case, GEMDOCK produced 800 solutions in one generation and was terminated after it exhausted 48,000 solutions for each compound in the screening set.

The screening quality of the docking methods using energy-based scoring functions alone is often influenced by the structure of the ligand screened (e.g., the numbers of charged and polar atoms). These methods are often biased toward charged polar compounds due to the pair-atom potentials of the electrostatic and hydrogen-bonding energies. In order to reduce this effect, GEMDOCK can evolve the pharmacological preferences from either a number of known active ligands or domain knowledge to take advantage of the similarities of putative ligands to those that are known to bind a protein's active site, thereby guiding the docking of the putative ligands [16]. Therefore, GEMDOCK is capable of using either a purely empirical scoring function [15] or a pharmacophore-based scoring function [16]. When GEMDOCK uses a pharmacophore-based scoring function, either certain known active ligands (more than two) or domain knowledge are required for the evolution of the pharmacological consensus. The empirical binding energy (*E_bind_*) is given as [15]:
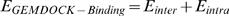
(1)where *E_inter_* and *E_intra_* are the intermolecular and intramolecular energies, respectively [15]. The pharmacophore-based energy function can be expressed as [16]:
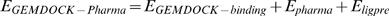
(2)where *E_GEMDOCK-Bind_* is the empirical binding energy defined in Equation (1), *E_pharma_* is the energy of the binding site pharmacophores (hot spots), and *E_ligpre_* is the penalty value when a ligand does not satisfy the ligand preferences [16]. *E_pharma_* and *E_ligpre_* are especially useful in the selection of active compounds from hundreds of thousands of non-active compounds by the exclusion of ligands that violate the characteristics of known active ligands (or domain knowledge). The pharmacophore-based interaction energy (*E_pharma_*) between the ligand and the protein is calculated with the assumption that the binding energies of all hot-spot atoms can be represented by the following equation [16]:
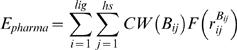
(3)where *CW*(*B_ij_*) is a pharmacological-weight function of a hot-spot atom *j* with interaction type *B_ij_*, 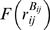 is as defined previously [15], *lig* is the number of heavy atoms in a screened ligand, and *hs* is the number of hot-spot atoms in the protein. The ligand preference (*E_ligpre_*) is the penalty value for the screened ligands that violate the electrostatic or hydrophilic constraints. *E_ligpre_* is given as [16]:
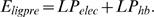
(4)where *LP_elec_* and *LP_hb_* are the penalties for the electrostatic (i.e., the number of charged atoms in a screened ligand) and hydrophilic (i.e., the fraction of polar atoms in a screened ligand) constraints, respectively.

### Plaque formation assay for the inhibitory effects of compounds on DV2 propagation

A local DV type 2 strain, PL046, was used to infect mosquito C6/36 cells for the production of DV type 2 virions. Mammalian BHK-21 host cells were cultured at 37°C with 5% CO_2_ in MEM medium (Gibco) supplemented with 0.22% sodium bicarbonate and 10% fetal bovine serum (FBS) (Gibco). C6/36 cells were grown at 28°C in MEM medium (Gibco) supplemented with 0.11% sodium bicarbonate and 10% FBS [23]. BHK-21 cells were plated at a density of 4×10^5^ cells per well in 6-well plates and incubated at 37°C with 5% CO_2_ for 48 hours. Different dilutions of the compounds were added to the 6-well plates followed by 0.5 mL of medium containing 200 PFUs of the DV type 2 PL046 strain per well. The mixtures were mixed gently and then incubated at 37°C with 5% CO_2_ for 1 hour. The supernatant in the culture was aspirated prior to the addition of a 1∶1 mixture of MEM medium:2% methylcellulose. The culture was then incubated at 37°C with 5% CO_2_ for 7 days. The medium was aspirated prior to fixation of the cells with 3.7% formaldehyde for 30 minutes. Then, the fixing solution was removed and the cells were stained with 1% crystal violet in 3.7% formaldehyde. Finally, the plates were washed with 3.7% formaldehyde prior to scoring of plaques [34].

## Supporting Information

Table S1 GEMDOCK cross-docking results of docking seven tetracycline-derivatives into five TetR protein structures and DV E protein
(0.02 MB PDF)

Table S2 Comparisons of the hydrogen bonds of five compounds between the dengue E protein and TetR protein
(0.02 MB PDF)

Figure S1 Docked conformations of the four tetracycline-derivatives. The two active compounds are rolitetracycline (blue) and doxycycline (green). The two inactive compounds are tetracycline (orange) and oxytetracycline (red). The inhibitory compounds are docked in positions leaning on the residues of the 48–52 stretch, of which the conformations in prefusion and postfusion states are very different. Residues affecting the pH threshold of fusion are indicated by numbers.
(0.07 MB PDF)

Figure S2 The surfaces and the docked conformations of the four tetracycline-derivatives on TetR protein and DV E protein according to GEMDOCK. (A) TetR protein (PDB code 2TRT); (B) DV E protein (PDB code 1OKE). The surfaces and sizes of the binding sites of these two proteins are similar. In addition, docked conformations of the four tetracycline-derivatives in these two proteins are also similar.
(0.09 MB PDF)

Appendix S1 Appendix A: The top 173 compounds of GEMDOCK by screening the CMC database.
(0.18 MB PDF)

## References

[1] Hayes EB, Gubler DJ (2006). West Nile virus: Epidemiology and clinical features of an emerging epidemic in the United States.. Annual Review of Medicine.

[2] Brinton MA (2002). The molecular biology of West Nile virus: A new invader of the Western hemisphere.. Annual Review of Microbiology.

[3] Leyssen P, Clercq ED, Neyts J (2000). Perspectives for the treatment of infections with Flaviviridae.. Clinical Microbiology Reviews.

[4] Gubler DJ, Gubler DJ (1997). Dengue and dengue hemorrhagic hever: its history and resurgence as a global public health problem.. New York: CAB International.

[5] Modis Y, Ogata S, Clements D, Harrison SC (2003). A ligand-binding pocket in the dengue virus envelope glycoprotein.. Proceedings of the National Academy of Sciences of the United States of America.

[6] Lyne PD (2002). Structure-based virtual screening: an overview.. Drug Discovery Today.

[7] Shoichet BK, McGovern SL, Wei B, Irwin J (2002). Lead discovery using molecular docking.. Current Opinion in Chemical Biology.

[8] Shoichet BK (2004). Virtual screening of chemical libraries.. Nature.

[9] Ghosh S, Nie AH, An J, Huang ZW (2006). Structure-based virtual screening of chemical libraries for drug discovery.. Current Opinion in Chemical Biology.

[10] Monath TP, Henize FX, Fields BN, Knipe DM, Howley PM (1996). Flaviviruses.. Fields virology, 3rd ed.

[11] Modis Y, Ogata S, Clements D, Harrison SC (2004). Structure of the dengue virus envelope protein after membrane fusion.. Nature.

[12] Lee E, Weir RC, Dalgarno L (1997). Changes in the dengue virus major envelope protein on passaging and their localization on the three-dimensional structure of the protein.. Virology.

[13] Rey FA, Heinz FX, Mandl C, Kunz C, Harrison SC (1995). The envelope glycoprotein from tick-borne encephalitis virus at 2 A resolution.. Nature.

[14] Yang J-M, Chen Y-F, Shen T-W, Kristal BS, Hsu DF (2005). Consensus scoring sriteria for Improving enrichment in virtual screening.. Journal of Chemical Information and Modeling.

[15] Yang J-M, Chen C-C (2004). GEMDOCK: a generic evolutionary method for molecular docking.. Proteins: Structure, Function, and Bioinformatics.

[16] Yang J-M, Shen T-W (2005). A pharmacophore-based evolutionary approach for screening selective estrogen receptor modulators.. Proteins: Structure, Function, and Bioinformatics.

[17] Yang J-M (2004). Development and evaluation of a generic evolutionary method for protein-ligand docking.. Journal of Computational Chemistry.

[18] Brodersen DE, Clemons JWM, Carter AP, Morgan-Warren RJ, Wimberly BT (2000). The structural basis for the action of the antibiotics tetracycline, pactamycin, and hygromycin B on the 30S ribosomal subunit.. Cell.

[19] Chopra I, Roberts M (2001). Tetracycline antibiotics: mode of action, applications, molecular biology, and epidemiology of bacterial resistance.. Microbiology and Molecular Biology Reviews.

[20] Shen M, LeTiran A, Xiao Y, Golbraikh A, Kohn H (2002). Quantitative structure-activity relationship analysis of functionalized amino acid anticonvulsant agents using k nearest neighbor and simulated annealing PLS methods.. Journal of Medicinal Chemistry.

[21] Jain AJ (2003). Surflex: fully automatic flexible molecular docking using a molecular similarity-based search engine.. Journal of Medicinal Chemistry.

[22] Beasley DWC, Aaskov JG (2001). Epitopes on the dengue 1 virus envelope protein recognized by neutralizing IgM monoclonal antibodies.. Virology.

[23] Hurrelbrink RJ, McMinn PC (2001). Attenuation of Murray Valley encephalitis virus by site-directed mutagenesis of the hinge and putative receptor-binding regions of the envelope protein.. Journal of Virology.

[24] Guirakhoo F, Zhang Z, Myers G, Johnson BW, Pugachev K (2004). A single amino acid substitution in the envelope protein of chimeric yellow fever-dengue 1 vaccine virus reduces neurovirulence for suckling mice and viremia/viscerotropism for monkeys.. Journal of Virology.

[25] Serafin IL, Aaskov JG (2001). Identification of epitopes on the envelope (E) protein of dengue 2 and dengue 3 viruses using monoclonal antibodies.. Archives of Virology.

[26] Monath TP, Arroyo J, Levenbook I, Zhang ZX, Catalan J (2002). Single mutation in the flavivirus envelope protein hinge region increases neurovirulence for mice and monkeys but decreases viscerotropism for monkeys: relevance to development and safety testing of live, attenuated vaccines.. Journal of Virology.

[27] Connell SR, Tracz DM, Nierhaus KH, Taylor DE (2003). Ribosomal protection proteins and their mechanism of tetracycline resistance.. Antimicrobial Agents and Chemotherapy.

[28] Orth P, Schnappinger D, Hillen W, Saenger W, Hinrichs W (2000). Structural basis of gene regulation by the tetracycline inducible Tetrepressor–operator system.. Nature.

[29] Laurie AT, Jackson RM (2005). Q-SiteFinder: an energy-based method for the prediction of protein-ligand binding sites.. Bioinformatics.

[30] Jones G, Willett P, Glen RC, Leach AR, Taylor R (1997). Development and validation of a genetic algorithm for flexible docking.. Journal of Molecular Biology.

[31] Kramer B, Rarey M, Lengauer T (1999). Evaluation of the flexX incremental construction algorithm for protein-ligand docking.. Proteins: Structure, Function, and Bioinformatics.

[32] Bissantz C, Folkers G, Rognan D (2000). Protein-based virtual screening of chemical databases. 1. Evaluation of different docking/scoring combinations.. Journal of Medical Chemistry.

[33] Yang J-M, Shen T-W, Chen Y-F, Chiu Y-Y (2004). An evolutionary approach with pharmacophore-based scoring functions for virtual database screening.. Lecture Notes in Computer Science.

[34] Chiu M-W, Yang Y-L (2003). Blocking the dengue virus 2 infections on BHK-21 cells with purified recombinant dengue virus 2 E protein expressed in Escherichia coli.. Biochemical and Biophysical Research Communications.

