# Supplementary material for: Combinatorial Computational Approaches to Identify Tetracycline Derivatives as Flavivirus Inhibitors
Source: PLoS One. 2007 May 9;2(5):e428. doi: 10.1371/journal.pone.0000428 (PMC1855430; doi:10.1371/journal.pone.0000428)
Supplement: Table S1 — GEMDOCK cross-docking results of docking seven tetracycline-derivatives into five TetR protein structures and DV E protein (0.02 MB PDF) [file pone.0000428.s001.pdf]

Table S1. GEMDOCK cross-docking results of docking seven tetracycline-derivatives into five TetR protein structures and DV E protein

protein \ ligand	Binding site volumn (Å^3) [a]	1ORK-atc [b]	2TCT-ctc	2TRT-tet	Tetracycline	Oxytetracycline	Rolitetracycline	Doxycycline
1BJ0-ctc [b] (TetR)	359	7.372 [c]	7.65	7.154	7.116	3.475	1.048	7.393
1DU7-ctc (TetR)	450	5.336	1.789	1.776	3.193	3.199	1.414	3.955
1ORK-atc (TetR)	383	1.4	1.568	0.788	2.905	3.108	1.017	3.703
2TCT-ctc (TetR)	495	5.175	1.863	0.721	1.801	1.745	1.598	3.650
2TRT-tet (TetR)	302	5.75	0.469	0.398	6.631	6.588	1.274	3.559
1OKE-BOG (E)	481	Inactive position [d]	Active position [e]	Inactive position	Inactive position	Inactive position	Active position [e]	Active position [e]

[a] Calculated by the tool q-sitefinder.
[b] The first four letters denote PDB code and latter three codes present ligand name in a complex.
[c] The RMSD value of the common ligand heavy atoms between the docked conformation and the crystal structure.
[d] The docked position is inside the binding site of E protein (see Figures 5C and 4D).
[e] The docked position is at the outlet of binding pocket and the structure extends into pocket (see Figures 5A and 4A)
