# Supplementary material for: Combinatorial Computational Approaches to Identify Tetracycline Derivatives as Flavivirus Inhibitors
Source: PLoS One. 2007 May 9;2(5):e428. doi: 10.1371/journal.pone.0000428 (PMC1855430; doi:10.1371/journal.pone.0000428)
Supplement: Table S2 — Comparisons of the hydrogen bonds of five compounds between the dengue E protein and TetR protein (0.02 MB PDF) [file pone.0000428.s002.pdf]

Table S2. Comparisons of the hydrogen bonds of five compounds between the dengue E protein and TetR protein

	DV E protein [a]				TetR protein [b]
	Tetracycline	Oxytetracycline	Rolitetracycline	Doxycycline	Chlortetracycline
Number of Hydrogen Bonds	7	9	8	8	6
Backbone	4	5	6	5	1
Side chain	3	4	2	3	5
Hydrogen Bonding to Backbone	Ala50O[c]-4N[d], 3.59[e]<br>Phe279O-10OH, 2.61<br>Phe279O-11O, 3.46<br>Thr48O-12αOH, 2.37	Thr48O-4N, 3.62<br>Ala50N-4αOH, 3.01<br>Thr48O-4αOH, 3.14<br>Thr48O-12OH, 2.33<br>Phe279O-11O, 2.62	Ala50N-1O, 2.77<br>Thr48O-2N, 3.17<br>Ala50O-10OH, 3.49<br>Ala50O-11O, 3.22<br>Ala50O-12OH, 2.97<br>Ala50N-12O, 3.16	Thr48O-1O, 3.62<br>Ala50N-1O, 2.76<br>Ala50N-12OH, 3.20<br>Ala50N-12αOH, 3.09<br>Thr48O-12αOH, 3.29	Thr103O-10OH, 3.64
Hydrogen Bonding to Side Chain	Gln271OE-2N, 2.62<br>Gln200NE-3OH, 2.67<br>Thr280OG-11O, 2.68	Gln271OE-2N, 2.89<br>Gln200NE-3OH, 3.33<br>Thr280OG-11O, 3.01<br>Thr280OG-12OH, 2.92	Gln200NE-6OH, 3.06<br>Glu49OE2-12αOH, 3.20	Gln200NE-6OH, 3.14<br>Glu49OE2-10OH, 3.14<br>Glu49OE2-11O, 3.57	Gln116NE2-2O, 3.27<br>His64NE2-3OH, 2.71<br>Asn82ND2-3OH, 2.82<br>Asn82OD2-4N, 2.70<br>His100NE2-12OH, 2.92

[a] The docked conformations against DV E protein (PDB entry 1OKE[3]).

[b] Tet-repressor protein (PDB entry is 2TRT [33]).

[c] Atom name of a residue and the number denotes the residue number in the target protein.

[d] Atom name of a compound and the number denotes the atom number in a compound.

[e] Distance of a pair of atoms forming a hydrogen bond between the protein and ligand.
