# Supplementary material for: Combinatorial Computational Approaches to Identify Tetracycline Derivatives as Flavivirus Inhibitors
Source: PLoS One. 2007 May 9;2(5):e428. doi: 10.1371/journal.pone.0000428 (PMC1855430; doi:10.1371/journal.pone.0000428)
Supplement: Figure S1 — Docked conformations of the four tetracycline-derivatives. The two active compounds are rolitetracycline (blue) and doxycycline (green). The two inactive compounds are tetracycline (orange) and oxytetracycline (red). The inhibitory compounds are docked in positions leaning on the residues of the 48–52 stretch, of which the conformations in prefusion and postfusion states are very different. Residues affecting the pH threshold of fusion are indicated by numbers. (0.07 MB PDF) [file pone.0000428.s003.pdf]

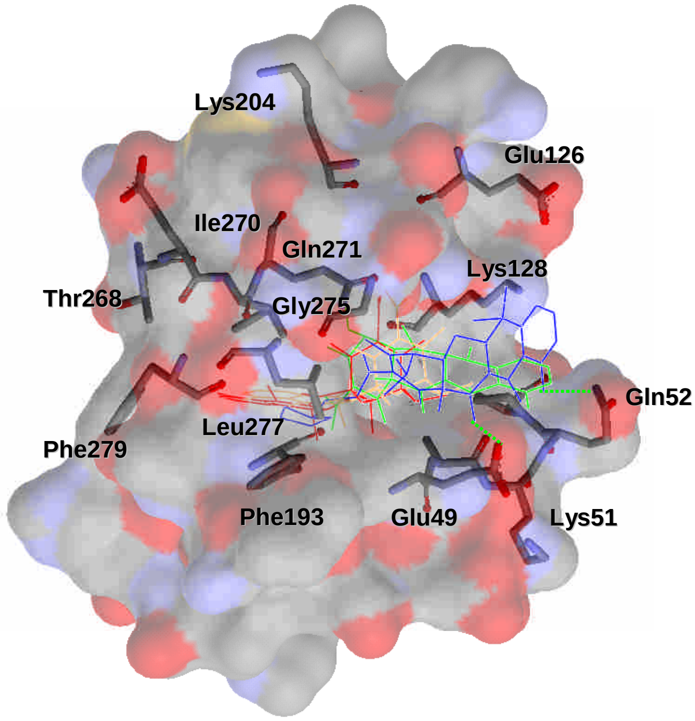

Figure S1. Docked conformations of the four tetracycline-derivatives. The two active compounds are rolitetracycline (blue) and doxycycline (green). The two inactive compounds are tetracycline (orange) and oxytetracycline (red). The inhibitory compounds are docked in positions leaning on the residues of the 48-52 stretch, of which the conformations in prefusion and postfusion states are very different. Residues affecting the pH threshold of fusion are indicated by numbers.
