# Supplementary material for: Combinatorial Computational Approaches to Identify Tetracycline Derivatives as Flavivirus Inhibitors
Source: PLoS One. 2007 May 9;2(5):e428. doi: 10.1371/journal.pone.0000428 (PMC1855430; doi:10.1371/journal.pone.0000428)
Supplement: Figure S2 — The surfaces and the docked conformations of the four tetracycline-derivatives on TetR protein and DV E protein according to GEMDOCK. (A) TetR protein (PDB code 2TRT); (B) DV E protein (PDB code 1OKE). The surfaces and sizes of the binding sites of these two proteins are similar. In addition, docked conformations of the four tetracycline-derivatives in these two proteins are also similar. (0.09 MB PDF) [file pone.0000428.s004.pdf]

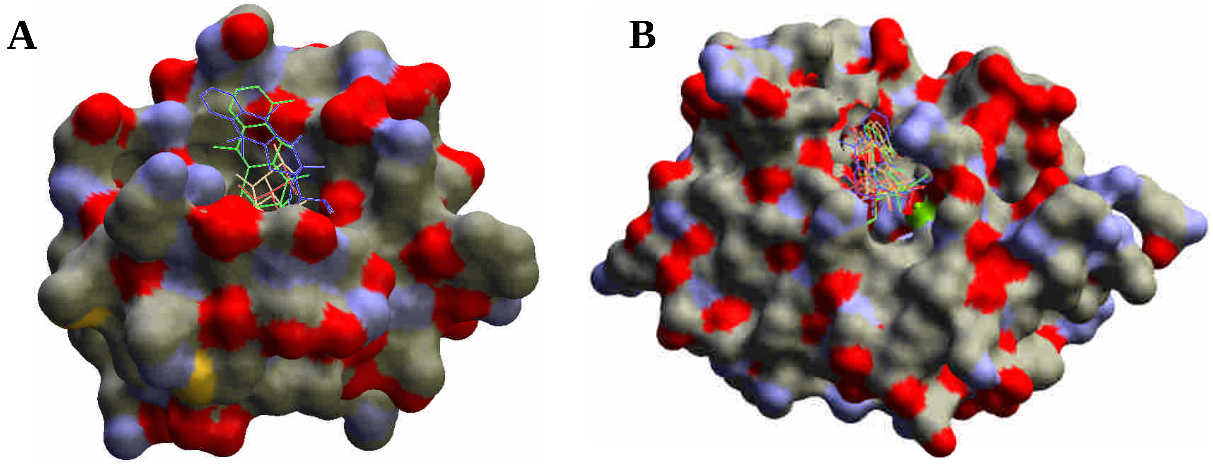

Figure S2. The surfaces and the docked conformations of the four tetracycline-derivatives on TetR protein and DV E protein according to GEMDOCK. (A) TetR protein (PDB code 2TRT); (B) DV E protein (PDB code 1OKE). The surfaces and sizes of the binding sites of these two proteins are similar. In addition, docked conformations of the four tetracycline-derivatives in these two proteins are also similar.
