# Supplementary material for: Combinatorial Computational Approaches to Identify Tetracycline Derivatives as Flavivirus Inhibitors
Source: PLoS One. 2007 May 9;2(5):e428. doi: 10.1371/journal.pone.0000428 (PMC1855430; doi:10.1371/journal.pone.0000428)
Supplement: Appendix S1 — Appendix A: The top 173 compounds of GEMDOCK by screening the CMC database. (0.18 MB PDF) [file pone.0000428.s005.pdf]

Appendix A: The top 173 compounds of GEMDOCK by screening the CMC database

Molecular structure	MDL number	molecular weight	Molecular Name	Rank	Energy
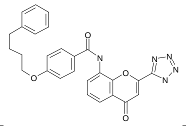	MCMC00006316	481.515	PRANLUKAST	1	-131.749
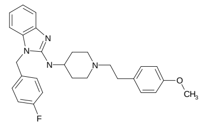	MCMC00004876	458.584	ASTEMIZOLE	2	-125.721
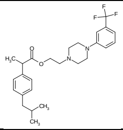	MCMC00005482	462.56	FRABUPROFEN	3	-124.395
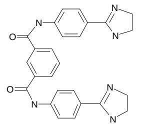	MCMC00002249	452.52	ISOTIC	4	-123.209
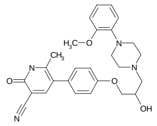	MCMC00005714	474.565	SATERINONE	5	-121.472
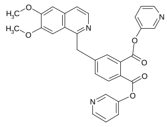	MCMC00001853	521.5341	NICEVERINE	6	-121.194
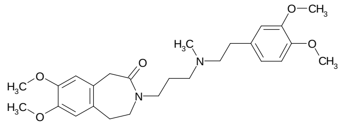	MCMC00005922	456.587	ZATEBRADINE	7	-120.367
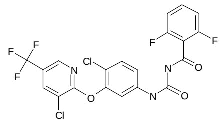	MCMC00006275	506.219	FLUAZURON	8	-119.333
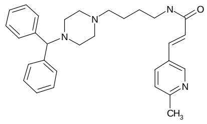	MCMC00007477	468.648	TAGORIZINE	9	-119.226
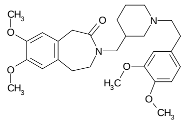	MCMC00006040	482.625	CILOBRADINE	10	-118.452
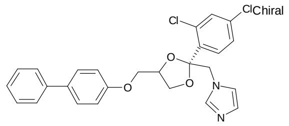	MCMC00004528	481.377	DOCONAZOLE	11	-118.317
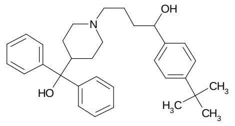	MCMC00004032	471.689	TERFENADINE	12	-112.280
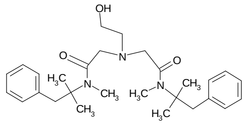	MCMC00000751	467.657	OXETHAZAINE	13	-111.531
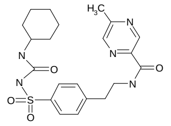	MCMC00003498	445.544	GLIPIZIDE	14	-109.651

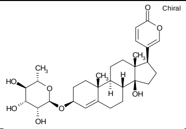	MCMC00007049	530.664	PROSCILLARIDIN	15	-105.257
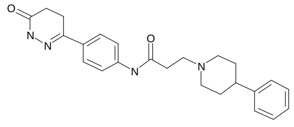	MCMC00005611	404.516	ALTAPIZONE	16	-96.518
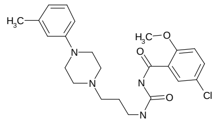	MCMC00004197	444.966	CILTOPRAZINE	17	-96.314
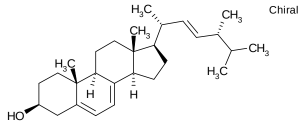	MCMC00006947	396.662	ERGOSTEROL	18	-96.032
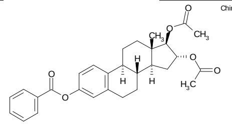	MCMC00010202	476.566	HOLIN-DEPOT	19	-95.330
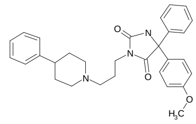	MCMC00004342	483.616	ROPITOIN	20	-95.185
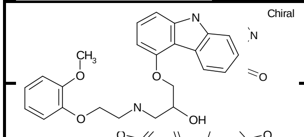	MCMC00007319	470.696	TRICAPRILIN	21	-94.863
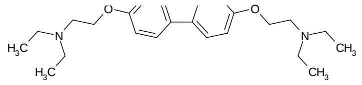	MCMC00003454	410.561	TILORONE	22	-94.706
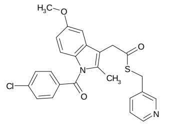	MCMC00005257	464.975	PIMETACIN	23	-94.512
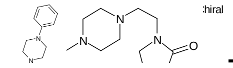	MCMC00003427	437.544	ALPERTINE	24	-94.142
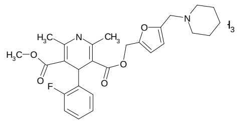	MCMC00006442	482.557	SAGANDIPINE	25	-93.803
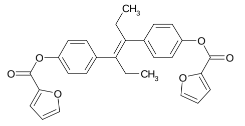	MCMC00001304	456.5	FUROSTILBESTROL	26	-93.700
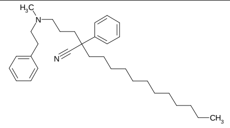	MCMC00005430	460.753	RONIPAMIL	27	-93.567
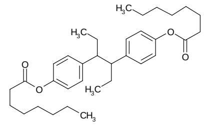	MCMC00010262	522.765	HEXESTROL DICAPRYLATE	28	-93.513
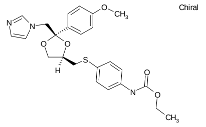	MCMC00006126	469.559	ERBULOZOLE	29	-93.366
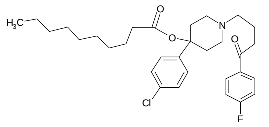	MCMC00005054	530.129	HALOPERIDOL DECANOATE	30	-93.054

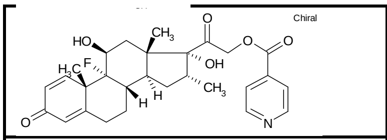	MCMC00010186	497.56	DEXAMETHASONE ISONICOTINATE	31	-92.851
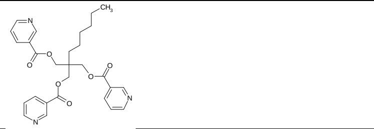	MCMC00002512	505.576	HEPRONICATE	32	-92.605
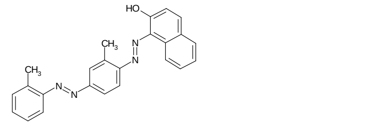	MCMC00000488	380.453	SCARLET RED	33	-92.555
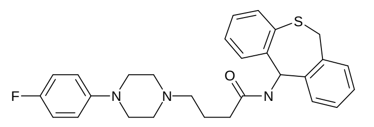	MCMC00006472	475.633	MONATEPIL	34	-92.543
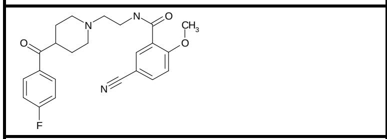	MCMC00005627	409.464	PRIDEPERONE	35	-92.288
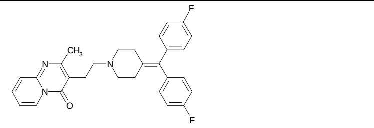	MCMC00005508	471.555	SEGANSERIN	36	-91.554
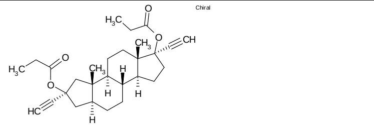	MCMC00010192	438.604	ANORDRIN	37	-91.336
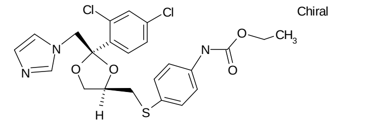	MCMC00005412	508.424	TUBULOZOLE	38	-91.198
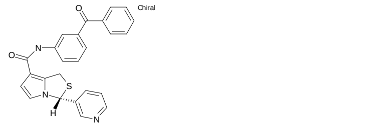	MCMC00006389	425.513	TULOPAFANT	39	-91.188
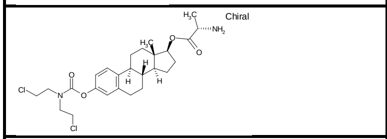	MCMC00007524	511.494	ALESTRAMUSTINE	40	-90.837
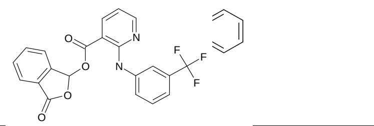	MCMC00005219	399.494	ANSOXETINE	41	-90.581
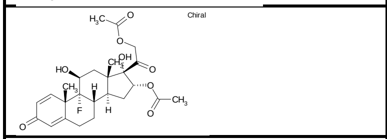	MCMC00006975	478.519	TRIAMCINOLONE DIACETATE	42	-90.375
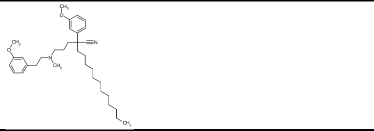	MCMC00005367	520.806	ANIPAMIL	43	-90.314
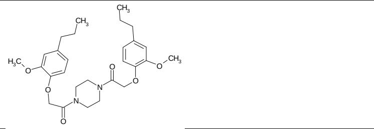	MCMC00000901	498.625	SIMETRIDE	44	-90.120
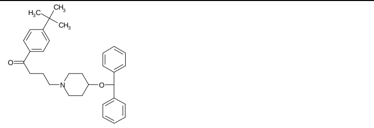	MCMC00005577	469.673	EBASTINE	45	-89.341

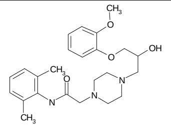	MCMC00005630	427.548	RANOLAZINE	46	-89.207
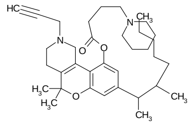	MCMC00004792	548.816	NABITAN	47	-89.134
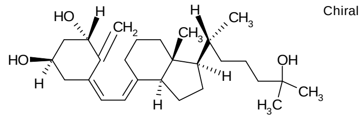	MCMC00003603	416.642	CALCITRIOL	48	-88.780
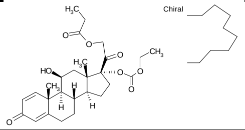	MCMC00005044	488.573	PREDNICARBATE	49	-88.760
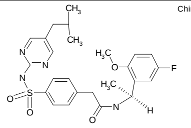	MCMC00003720	500.592	GLIFLUMIDE	50	-88.262
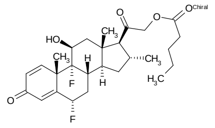	MCMC00010176	478.572	DIFLUCORTOLONE VALERATE	51	-87.957
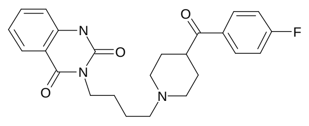	MCMC00005490	423.492	BUTANSERIN	52	-87.587
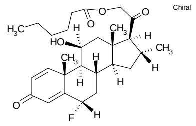	MCMC00000936	474.609	FLUOCORTOLONE CAPROATE	53	-87.124
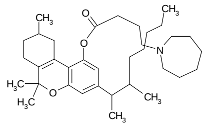	MCMC00004449	537.834	NABAZENIL	54	-86.942
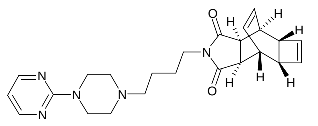	MCMC00006380	419.526	ZALOSPIRONE	55	-86.847
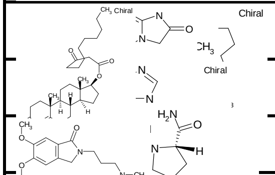	MCMC00006119	464.56	TRELNARIZINE	56	-86.544
	MCMC00006369	421.903	MOSAPRIDE	57	-86.497
	MCMC00005183	428.533	FALIPAMIL	58	-86.480
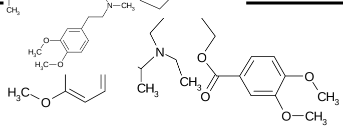	MCMC00002025	429.561	MEBEVERINE	59	-86.422
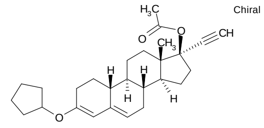	MCMC00001905	408.578	QUINGESTANOL ACETATE	60	-86.383
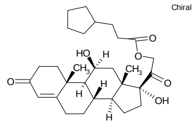	MCMC00001185	486.645	HYDROCORTISONE CYPIONATE	61	-86.350
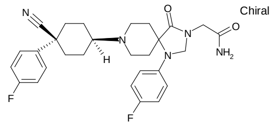	MCMC00005233	507.582	ICOSPIRAMIDE	62	-86.277

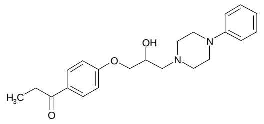	MCMC00010062	368.474	CENTPROPAZINE	63	-86.219
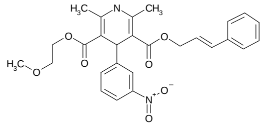	MCMC00006473	492.533	CILNIDIPINE	64	-86.174
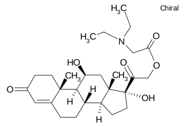	MCMC00000380	475.622	HYDROCORTAMATE	65	-85.929
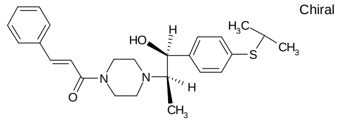	MCMC00005434	424.606	SUNAGREL	66	-85.252
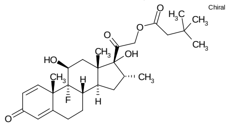	MCMC00010194	490.608	DEXAMETHASONE TERT.-BUTYLACETATE	67	-85.216
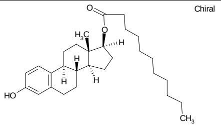	MCMC00002001	440.664	ESTRADIOL UNDECYLATE	68	-85.121
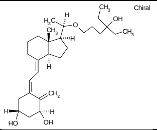	MCMC00007647	460.694	LEXACALCITOL	69	-85.021
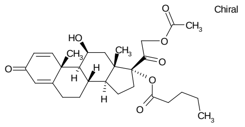	MCMC00006751	486.601	PREDNISOLONE VALERATE ACETATE	70	-84.715
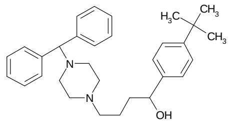	MCMC00005340	456.677	TRENIZINE	71	-84.591
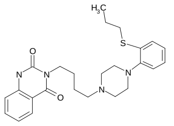	MCMC00004119	452.623	TIOPERIDONE	72	-84.396
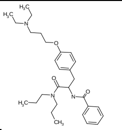	MCMC00004404	481.684	SEVOPRAMIDE	73	-84.342
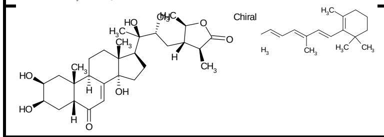	MCMC00010427	520.6589	CYASTERONE	74	-84.289
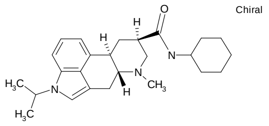	MCMC00006419	393.577	AMESERGIDE	75	-84.195
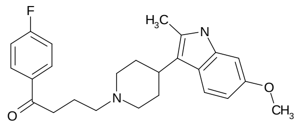	MCMC00004095	408.521	MINDOPERONE	76	-83.876
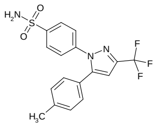	MCMC00008129	381.377	CELECOXIB	77	-83.840

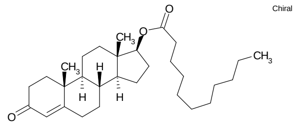	MCMC00010234	456.706	TESTOSTERONE UNDECANOATE	78	-83.790
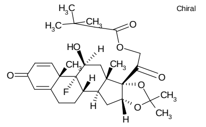	MCMC00002315	532.645	TRIAMCINOLONE HEXACETONIDE	79	-83.450
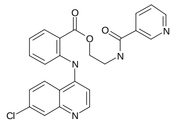	MCMC00004679	446.897	NICAFENINE	80	-82.912
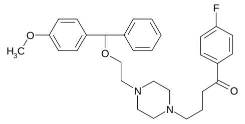	MCMC00004737	490.623	MOBENZOXAMINE	81	-82.904
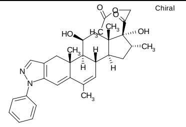	MCMC00001576	530.6609	CORTIVAZOL	82	-82.756
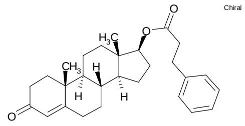	MCMC00010189	420.589	TESTOSTERONE PHENYLPROPIONATE	83	-82.657
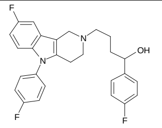	MCMC00004941	450.508	FLUTROLINE	84	-82.602
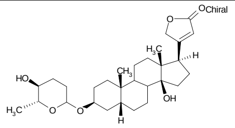	MCMC00003632	488.661	RAMNODIGIN	85	-82.503
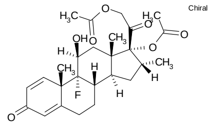	MCMC00006567	476.538	DEXAMETHASONE ACETATE	86	-82.145
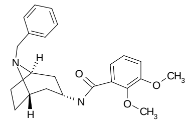	MCMC00005125	380.485	TROPAPRIDE	87	-81.839
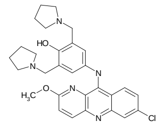	MCMC00010201	518.058	BENZONAPHTHYRIDINE 7351	88	-81.678
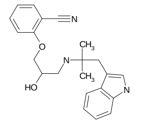	MCMC00004956	363.463	BUCINDOLOL	89	-81.493
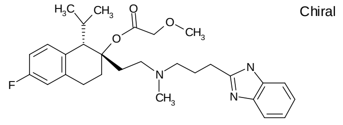	MCMC00007471	495.643	MIBEFRADIL	90	-80.946
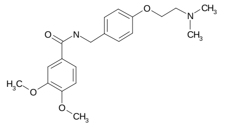	MCMC00006424	358.441	ITOPRIDE	91	-80.878
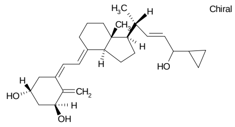	MCMC00006060	412.61	CALCIPOTRIOL	92	-80.817

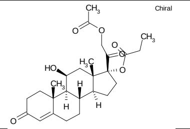	MCMC00005053	460.563	HYDROCORTISONE ACEPONATE	93	-80.780
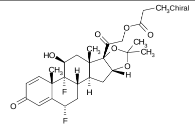	MCMC00007244	508.565	PROCINONIDE	94	-80.766
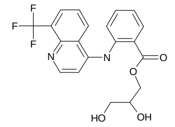	MCMC00003287	406.365	FLOCTAFENINE	95	-80.625
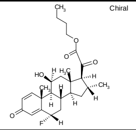	MCMC00003935	446.555	FLUOCORTIN BUTYL	96	-79.919
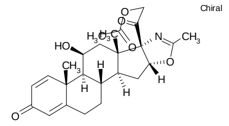	MCMC00002786	441.521	DEFLAZACORT	97	-79.318
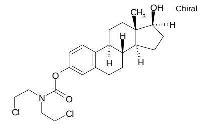	MCMC00001904	440.408	ESTRAMUSTINE	98	-77.901
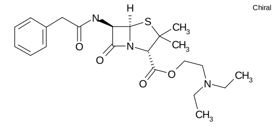	MCMC00010073	433.57	PENETHAMATE HYDROIODIDE	99	-77.861
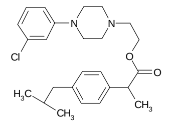	MCMC00005635	429.007	LOBUPROFEN	100	-77.750
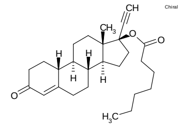	MCMC00010178	410.594	NORETHINDRONE ENANTHATE	101	-77.706
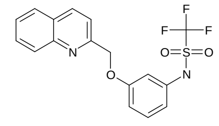	MCMC00006056	382.364	RITOLUKAST	102	-77.603
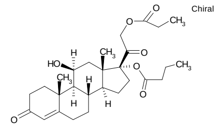	MCMC00005892	488.617	HYDROCORTISONE BUTEPRATE	103	-76.851
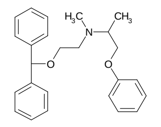	MCMC00004732	375.515	PRENOVERINE	104	-76.602
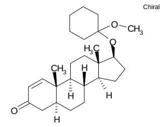	MCMC00002535	400.599	MESABOLONE	105	-75.995
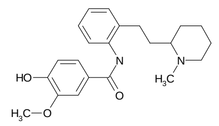	MCMC00005916	368.48	MODECAINIDE	106	-75.673
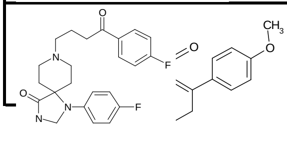	MCMC00004265	413.471	FLUSPIPERONE	107	-75.470

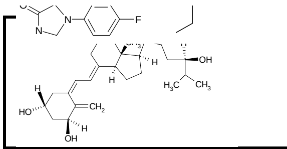	MCMC00006240	416.65	TACALCITOL	108	-75.311
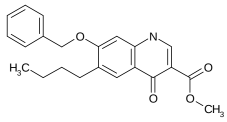	MCMC00002750	365.433	NEQUINATE	109	-74.676
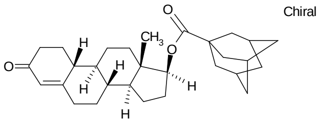	MCMC00001669	436.632	BOLMANTALATE	110	-74.639
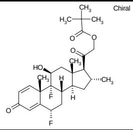	MCMC00007170	478.582	DIFLUCORTOLONE PIVALATE	111	-74.482
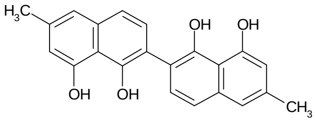	MCMC00010046	346.38	DIOSPYROL	112	-74.459
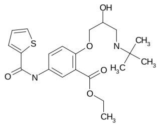	MCMC00005556	420.532	TIENOXOLOL	113	-74.035
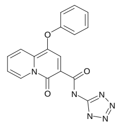	MCMC00006202	348.321	QUINOTOLAST	114	-73.874
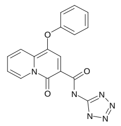	MCMC00006202	348.321	QUINOTOLAST	115	-73.874
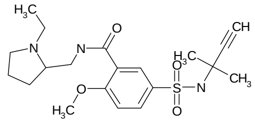	MCMC00004894	407.536	TINISULPRIDE	116	-73.656
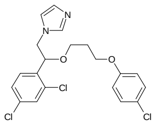	MCMC00004952	425.746	ZOFICONAZOLE	117	-73.571
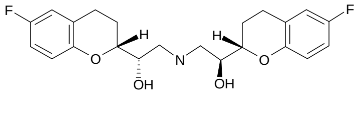	MCMC00005680	405.439	NEBIVOLOL	118	-73.196
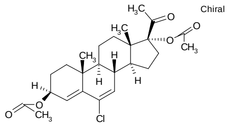	MCMC00001911	448.984	CLOGESTONE ACETATE	119	-73.124
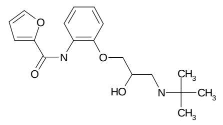	MCMC00005103	332.403	ANCAROLOL	120	-72.912
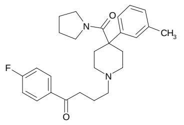	MCMC00010171	436.568	MEPERIDIDE	121	-72.613
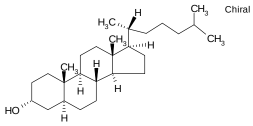	MCMC00006715	388.675	EPIDIHYDROCHOLESTERIN	122	-72.199

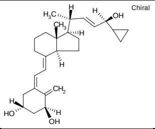	MCMC00006371	412.618	CALCIPOTRIENE	123	-72.013
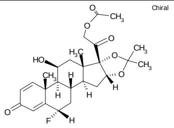	MCMC00002169	476.538	FLUNISOLIDE ACETATE	124	-71.378
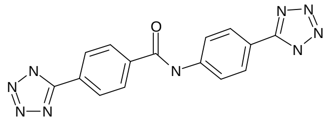	MCMC00006478	333.315	ANDOLAST	125	-70.674
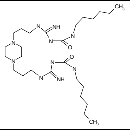	MCMC00004882	538.786	IPEXIDINE	126	-69.594
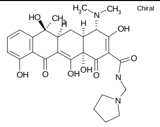	MCMC00007079	527.579	ROLITETRACYCLIN E	127	-69.574
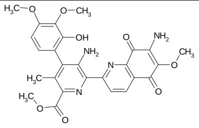	MCMC00001958	520.503	METHYLSTREPTONI GRIN	128	-66.946
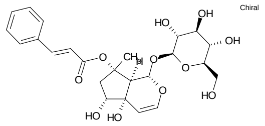	MCMC00010126	494.49	HARPAGOSIDE	129	-66.708
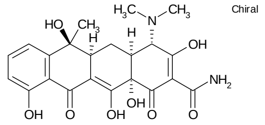	MCMC00006963	444.445	TETRACYCLINE	130	-60.419
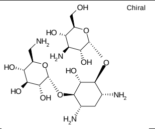	MCMC00006958	484.508	KANAMYCIN	131	-53.576
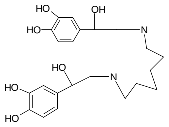	MCMC00001935	420.51	HEXOPRENALINE	132	-52.259
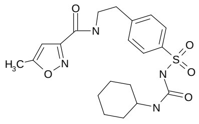	MCMC00003320	434.518	GLISOLAMIDE	133	-51.420
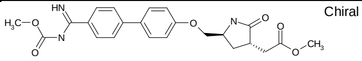	MCMC00007873	439.465	LEFRADAFIBAN	134	-50.403
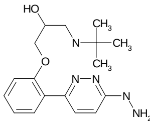	MCMC00004492	331.421	PRIZIDILOL	135	-50.205
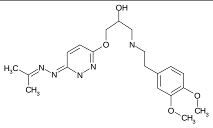	MCMC00005150	403.485	DRAMEDILOL	136	-47.967
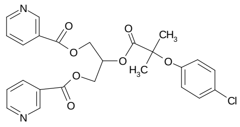	MCMC00004883	498.924	BINIFIBRATE	137	-46.008

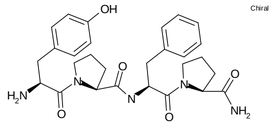	MCMC00010188	521.615	MORPHICEPTIN	138	-45.856
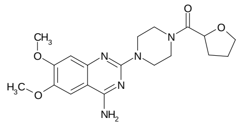	MCMC00004658	387.442	TERAZOSIN	139	-45.411
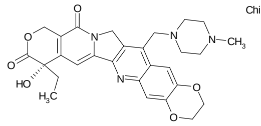	MCMC00007618	518.567	GI147211	140	-44.963
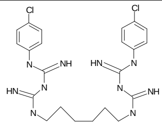	MCMC00000120	505.457	CHLORHEXIDINE	141	-44.314
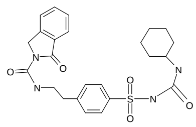	MCMC00004946	484.579	GLISINDAMIDE	142	-43.950
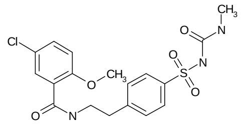	MCMC00004132	425.894	GLICONDAMIDE	143	-43.451
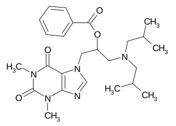	MCMC00007448	469.589	DIISOBUTYLAMINO BENZOYLOXYPROP YL THEOPHYLLINE	144	-43.293
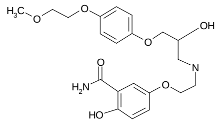	MCMC00005147	420.467	TRIGEVOLOL	145	-42.905
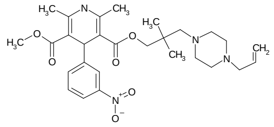	MCMC00006786	526.638	IGANIDIPINE	146	-42.714
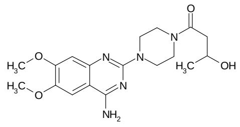	MCMC00006353	375.431	NELDAZOSIN	147	-41.933
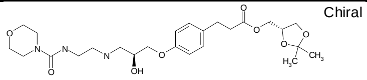	MCMC00007871	509.596	LANDIOLOL	148	-41.717
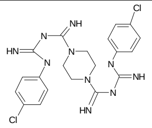	MCMC00002335	475.387	PICLOXYDINE	149	-40.938
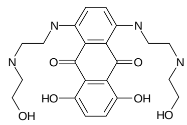	MCMC00004733	444.492	MITOXANTRONE	150	-40.259
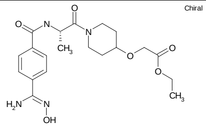	MCMC00007926	420.463	SIBRAFIBAN	151	-38.854
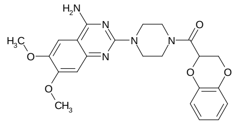	MCMC00005060	451.486	DOXAZOSIN	152	-38.541

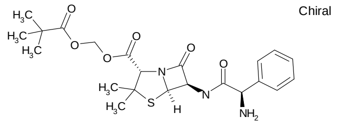	MCMC00003657	463.552	PIVAMPICILLIN	153	-37.287
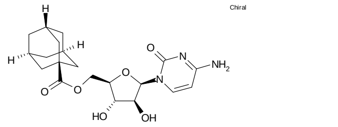	MCMC00009952	405.448	ADAMANTOYLCYTA RABINE	154	-37.025
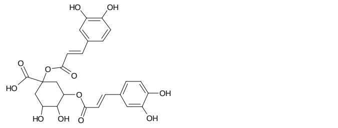	MCMC00001729	516.463	CYNARINE	155	-36.837
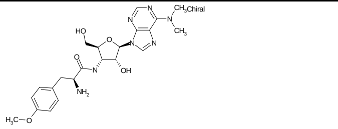	MCMC00006935	471.52	PUROMYCIN	156	-34.527
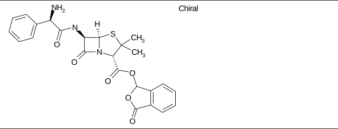	MCMC00003999	481.527	TAMAMPICILLIN	157	-34.508
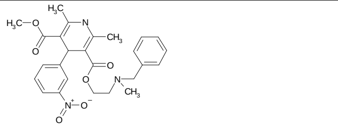	MCMC00004336	479.538	NICARDIPINE	158	-33.459
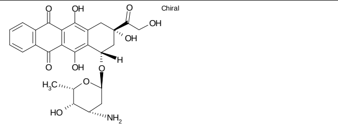	MCMC00004696	513.496	MEDORUBICIN	159	-31.643
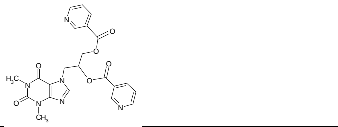	MCMC00003023	464.441	DINIPROFYLLINE	160	-30.049
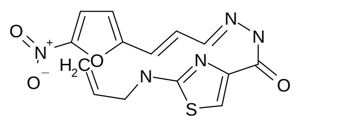	MCMC00004250	347.355	NIFURALIDE	161	-29.937
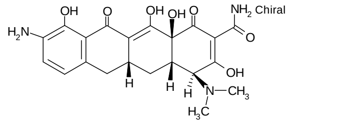	MCMC00002374	429.427	AMICYCLINE	162	-29.299
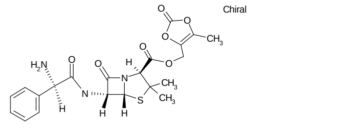	MCMC00005462	461.493	LENAMPICILLIN	163	-29.040
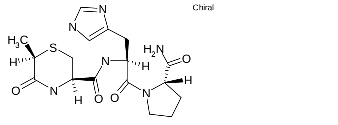	MCMC00005563	408.481	MONTIRELIN	164	-28.311
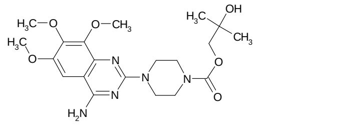	MCMC00003746	435.484	TRIMAZOSIN	165	-28.153
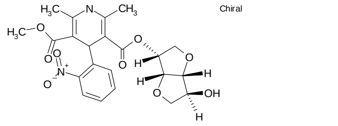	MCMC00005624	460.437	SORNIDIPINE	166	-26.193
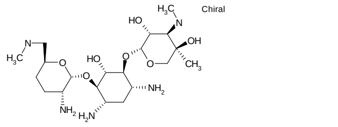	MCMC00004092	463.572	MICRONOMICIN	167	-25.181

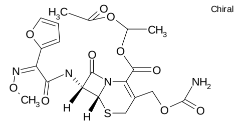	MCMC00004703	510.478	CEFUROXIME AXETIL	168	-21.350
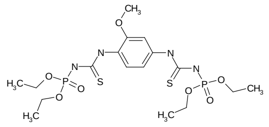	MCMC00004798	528.527	IMCARBOFOS	169	-19.970
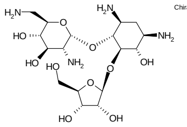	MCMC00003356	454.474	RIBOSTAMYCIN	170	-18.811
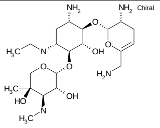	MCMC00004361	475.583	NETILMICIN	171	-18.155
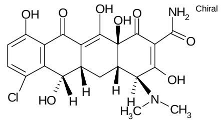	MCMC00000758	464.856	DEMECLOCYCLINE	172	-17.077
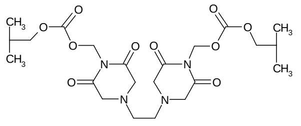	MCMC00005987	514.537	SOBUZOXANE	173	-11.418
